# Epidemiology of osteoporotic fracture in Kazakhstan and development of a country specific FRAX model

**DOI:** 10.1007/s11657-020-0701-3

**Published:** 2020-02-27

**Authors:** S. Issayeva, O. Lesnyak, A. Zakroyeva, B. Issayeva, D. Dilmanova, H. Johansson, E. Liu, M. Lorentzon, N.C. Harvey, E. McCloskey, J.A. Kanis

**Affiliations:** 1 Asfendiyarov National Medical University, 94, Tole Bi Street, Almaty, Kazakhstan 050000; 2 Mechnikov North West State Medical University, 41, Kirochnaya Street, 191015 St. Petersburg, Russia; 3 Ural State Medical University, 3, Repina Street, 620028 Yekaterinburg, Russia; 4 Mary McKillop Health Institute, Australian Catholic University, Melbourne, Australia; 5 Institute of Medicine, University of Gothenburg, Gothenburg, Sweden; 6 MRC Lifecourse Epidemiology Unit, University of Southampton, Southampton, UK; 7 Centre for Metabolic Bone Diseases, University of Sheffield, Sheffield, UK

**Keywords:** FRAX, Fracture probability, Epidemiology, Hip fracture, Forearm fracture, Humerus fracture, Kazakhstan

## Abstract

***Summary*:**

Retrospective and prospective population-based survey in a region of the Republic of Kazakhstan determined the incidence of fractures at the hip, proximal humerus and distal forearm. The hip fracture rates were used to create a FRAX® model to enhance fracture risk assessment in Kazakhstan.

**Objective:**

This paper describes the epidemiology of osteoporotic fractures in the Republic of Kazakhstan that was used to develop a country specific FRAX® tool for fracture prediction.

**Methods:**

We carried out a retrospective population-based survey in Taldykorgan in the Republic of Kazakhstan representing approximately 1% of the country’s population. Hip, forearm and humerus fractures were identified retrospectively in 2015 and 2016 from hospital registers and the trauma centre. Hip fractures were prospectively identified in 2017 from the same sources and additionally from primary care data. Age- and sex-specific incidence of hip fracture and national mortality rates were incorporated into a FRAX model for Kazakhstan. Fracture probabilities were compared with those from neighbouring countries having FRAX models.

**Results:**

The difference in hip fracture incidence between the retrospective and prospective survey indicated that approximately 25% of hip fracture cases did not come to hospital attention. The incidence of hip fracture applied nationally suggested that the estimated number of hip fractures nationwide in persons over the age of 50 years for 2015 was 11,690 and is predicted to increase by 140% to 28,000 in 2050. Hip fracture incidence was a good predictor of forearm and humeral fractures in men but not in women.

**Conclusion:**

The FRAX model should enhance accuracy of determining fracture probability among the Kazakh population and help guide decisions about treatment.

## Introduction

Osteoporosis is a common, chronic and costly condition; its only clinical consequence is fracture. In Europe, the annual cost of fractures associated with osteoporosis exceeded € 37 billion in 2010 [1], and disability due to osteoporosis was greater than that caused by any single cancer, with the exception of lung cancer and was comparable or greater than that lost to a variety of chronic noncommunicable diseases, such as rheumatoid arthritis, asthma and high blood pressure related to heart disease [2]. Fortunately, a wide variety of treatments is available that favourably affect bone mass and thereby decrease the risk of fractures associated with osteoporosis [3]. The use of such interventions by health care practitioners is assisted by instruments that assess patients’ fracture risk to optimize clinical decisions about prevention and treatment. The most widely used web-based tool FRAX® (https://www.sheffield.ac.uk/FRAX/) meets these requirements and computes the 10-year probability of fragility fractures based on several common clinical risk factors and optionally a DXA scan result [4, 5]. FRAX models are available for 66 countries in 2020 covering more than 80% of the world population at risk [6], and have been incorporated into more than 100 guidelines worldwide [7].

The availability of FRAX has stimulated studies that can be used for the generation of new FRAX models. Specific examples include Brazil, Mexico and Turkey [8]. The present study is a component part of the Multicenter Multinational population-based Study in Eurasian Countries (EVA study or ЭВА, in Russian). The broad aim of the study was to provide epidemiological information on fracture risk so that FRAX models could be created for Russia [9], Armenia [10], Belarus [11], Moldova [12], Kazakhstan and Uzbekistan. The present report describes the epidemiology of fractures at the hip, forearm and humerus in Kazakhstan and the generation of a country specific FRAX model.

## Methods

The Republic of Kazakhstan is the world’s largest landlocked country and the ninth largest in the world, with an area of 2,724,900 km^2^. Kazakhstan shares borders with Russia, China, Kyrgyzstan, Uzbekistan, Turkmenistan, and the Caspian Sea. In 2015 the population of Kazakhstan was 17.75 million and rose to 18.20 million in 2017 [13].

For the present study, Taldykorgan (Taldıqorğan), the administrative centre of Almaty Region of Kazakhstan, was chosen as the catchment area. Taldykorgan was selected because of its long distance from other major cities of the Republic and the availability of highly specialized orthopaedic care for all Taldykorgan residents. This minimized the possibility of residents seeking medical care for their fracture in neighbouring cities. Each individual in Kazakhstan has a unique digital code which permits the number of residents to be determined by region, age and sex, the precise number of inhabitants counting in any period of time [14]. The total catchment population of the regions was 165,296 representing 0.9% of the total population. The age, sex and ethnic distribution were very similar to that of the whole country. The ethnic distribution was Kazakhs (66.5%), Russian (20.6%) and other ethnicities (12.9%) [14].

The study was organized in two phases. The first was a retrospective survey from 1 January 2015 to 31 December 2016 which captured data on data on fractures at the hip (ICD-10 codes S72.0, S72.1, S72.2), distal forearm (S52.5, S52.6) and proximal humerus (S 42.2). The second phase was a prospective survey from 1 March 2017 to 28 February 2018 that acquired data on hip fracture alone.

In both phases, the medical records of all fractures in men and women aged 40 years or older were retrieved from the inpatient electronic health register (EHR) of the three hospitals in the area, the outpatient register of the City Trauma centre. In addition, refusals of hospitalization (formal documents) were examined from all the hospitals of the city. Only fractures validated by radiographs were included. To avoid double counting, further admissions for the same fracture site in the observation time were excluded. In some documents, fracture ICD-10 code was not specified. In such cases, radiographs were retrieved and fractures, if verified, were included in the database. Permanent residence in the region was a criterion for inclusion. All hip fracture cases were included irrespective of high or low energy trauma. We excluded pathological fractures attributable to cancer with metastases or to multiple myeloma.

The prospective study identified new cases of hip fractures using the same methodology as in the retrospective survey. In addition, data were gathered from the records of the emergency call centre, from the records of home visits to patients by orthopaedic doctors from the outpatient polyclinic, the records and outpatient electronic health records of all (32) primary care doctors in the city and two private primary health care centres to find additional non-hospitalized patients. These patients were examined at home, and the hip fracture was verified clinically, and where possible, by radiography.

Yearly incidence rates for fractures of the distal forearm and proximal humerus were estimated from the number of men and women in 5- or 10-year age intervals with at least one index fracture in 2015 and 2016 divided by the age- and sex-specific population at risk. In the case of hip fracture, the prospective study identified more men and women than the retrospective surveys of 2015 and 2016. For example, 65 hip fracture cases were identified in women in 2015 and 65 in 2016. In contrast, an additional 19 fractures were identified in 2017 (i.e. a total of 84 hip fractures). We assumed that a similar number of fractures (19) had been missed in 2015 and 2016 and uplifted the incidence rates in these years by 29% ((65 + 19)/65). In the case of men, the incidence was upward revised by 8%.

The adjusted age and sex-specific incidence in 2015–2017 was applied to the Kazakh population in 2015 to estimate the number of hip fractures nationwide. Additionally, future projections were estimated up to 2050 assuming that the age- and sex-specific incidence remained stable. Population demography was taken from the United Nations using the medium variant for fertility [15].

The adjusted data on hip fracture were used to construct the FRAX model. For other major osteoporotic fractures (clinical spine, forearm and humeral fractures), it was assumed that the age- and sex-specific ratios of these fractures to hip fracture, risk found in Sweden were comparable to those in Kazakhstan. This assumption has been used for many of the FRAX models with incomplete epidemiological information. Available information suggests that the age- and sex-stratified pattern of fracture is very similar in the Western world, Australia and Eastern Europe [12, 16–18]. In order to test this further, we compared the incidence of a forearm or humeral fracture observed in Kazakhstan with the incidence that would be predicted from the pattern of incidence in Malmo applied to the incidence of hip fracture in Kazakhstan. This assumes that the age- and sex-specific pattern of incidence of proximal humerus and forearm fracture (i.e. other major fractures, OMF) and the adjusted hip fracture (HF) in Kazakhstan are similar to that seen in Malmo [16]. Thus, for each age and sex,

$$ \frac{{\mathrm{HF}}_{\mathrm{Kazakhstan}}}{{\mathrm{HF}}_{\mathrm{Malmo}}}=\frac{{\mathrm{OMF}}_{\mathrm{Kazakhstan}}}{{\mathrm{OMF}}_{\mathrm{Malmo}}} $$

therefore,

$$ {\mathrm{OMF}}_{\mathrm{Kazakhstan}}=\frac{{\mathrm{HF}}_{\mathrm{Kazakhstan}}\times {\mathrm{OMF}}_{\mathrm{Malmo}}}{{\mathrm{HF}}_{\mathrm{Malmo}}} $$

From this, the incidence of a forearm or humerus fracture, estimated using the Malmo ratios, was compared with the empirical data from Kazakhstan from the ages of 50–90 years.

The development and validation of FRAX have been extensively described [4, 5]. The risk factors used were based on a systematic set of meta-analyses of population-based cohorts worldwide and validated in independent cohorts with over 1 million patient-years of follow-up. The construct of the FRAX model for Kazakhstan retained the beta coefficients of the risk factors in the original FRAX model with the incidence rates of hip fracture and mortality rates for Kazakhstan. National mortality rates used data from the World Health Organization for 2015 [19]. Ten-year fracture probabilities were compared to those of neighbouring countries where a FRAX model was available (China and Russia).

In order to compare Kazakh hip fracture probabilities with those of other regions of the world, the remaining lifetime probability of hip fracture from the age of 50 years was calculated for men and women, as described previously [20]. In the present analysis, values for Kazakhstan were compared with those of China (with and without inclusion of Hong Kong), Canada, Denmark, Finland, France, Hungary, Mexico, Moldova, Poland, Portugal, Russia, Spain, Sweden, Turkey, Ukraine, the UK and the USA.

## Results

A total of 1058 fractures were identified in individuals aged 40 years or more. These comprised 348 hip fractures (2015, 2016 and 2017), 174 humerus and 536 distal forearm fractures (2015 and 2016).

### Hip fracture

A total of 134 hip fractures were identified in men and 214 in women (female/male ratio 1.6). Below the age of 70 years, hip fractures were more common in men than in women (female/male ratio 0.8) but thereafter were more frequent in women (female/male ratio 3.1). The incidence of hip fracture increased with age in men and women, though more markedly in women (Table 1). Of the 348 cases of hip fractures, 82 cases formally (24%) refused hospital admission (27 men and 55 women). The cases that declined admission increased in frequency with age. Of the 266 patients admitted to the hospital, 200 (75%) underwent surgery. In total 43% of hip fracture cases were either untreated or managed conservatively.

**Table 1:** Population of the catchment area, number of hip fractures and annual incidence of hip fractures (rate/100,000) in men and in women from Taldykorgan, Kazakhstan by age for 2015, 2016 and 2017 combined

Age (years)	Population	Fractures^a^	Incidence/100,000^b^	95% CI
*Men*				
40–44	15,668	10	67	31–117
45–49	14,234	9	68	29–120
50–54	13,691	10	77	35–134
55–59	11,371	18	167	101–261
60–64	8377	18	225	127–353
65–69	6387	23	378	241–562
70–74	3279	8	254	105–481
75–79	2665	13	527	287–884
80–84	1323	10	780	362–1390
85–89	593	12	2078	1046–3605
90–94	211	3	1536	289–4156
95 +	150	0	–	–
40 +	77,949	134	181	152–213
*Women*				
40–44	19,142	5	31	8–61
45–49	17,874	4	271	6–57
50–54	17,099	6	42	17–84
55–59	15,231	14	109	60–176
60–64	11,984	16	158	89–247
65–69	10,058	25	292	193–418
70–74	5169	21	502	313–736
75–79	5665	44	925	692–1212
80–84	2833	38	1515	1096–2042
85–89	1360	31	2663	1867–3684
90–94	357	10	3207	1537–5514
95+	108	0	–	–
40+	106,880	214	236	207–266

^a^Unadjusted numbers

^b^Includes adjusted incidence for 2015 and 2016

### Forearm and humeral fractures

Fractures at the distal forearm were more frequent in women than in men (female/male ratio = 4.3). There was no clear age-dependent trend of incidence in women or men (Table 2).

**Table 2:** Number and annual incidence of forearm and humeral fractures (rate/100,000) in men and women in Taldykorgan, Kazakhstan by age for 2015 and 2016 combined

	Forearm			Humerus		
Age (years)	Fractures	Incidence	95% CI	Fractures	Incidence	95% CI
*Men*						
40–49	38	192	136–264	13	66	35–113
50–59	38	229	162–315	18	109	64–172
60–69	20	206	126–318	15	154	86–255
70–79	3	78	16–226	2	52	6–187
80–89	2	1658	20–598	0	–	–
90 +	0			1	424	8–2364
40 +	101	197	160–239	49	95	71–126
*Women*						
40–49	90	366	294–450	14	57	31–96
50–59	172	8046	689–934	35	164	114–228
60–69	97	6736	546–821	39	271	192–370
70–79	53	743	556–971	21	294	182–450
80–89	20	748	457–1156	16	599	342–972
90 +	3	952	196–2785	0	–	–
40 +	435	617	560–678	125	177	148–211

The annual incidence of proximal humerus fractures was lower in men than in women (female/male ratio = 2.6). Humeral fractures were less common than forearm fractures, and in women, increased with age.

### Fracture projections

Assuming that the fracture rates in Taldykorgan was representative for the whole country, and based on the UN estimates of Kazakh population for 2015, we estimated that the annual number of hip fractures in men and women age 50 years and older in Kazakhstan in 2015 was 11,690, comprising 3815 in men and 7875 fractures in women. The number of hip fractures is expected to increase progressively by calendar year with an increase of 140% by 2050 (Table 3). The increase in hip fracture numbers is particularly great in women (153% in women and 112% in men) due to the high age dependency of hip fracture incidence.

**Table 3:** Estimated total number of hip fractures (ICD-10 codes S72.0, S72.1 and S72.2) in men and in women age 50 years and older in 2015 projected up to 2050 in Kazakhstan

	2015	2020	2030	2040	2050
Men	3815	4298	5234	6645	8110
Women	7875	8653	11,293	15,837	19,938
Total	11,690	12,951	16,527	22,482	28,048
Increase (%)	–	11	41	92	140

### Fracture probability

In men, the incidence of forearm and humeral fractures was very similar to that predicted from the epidemiology of fracture in Malmo (Table 4). In women, however, the observed fracture rates exceeded those predicted from the Malmo ratios, in some cases significantly so (Table 4). Because of the discordance in the findings between men and women, the FRAX model was based on the data on hip fracture, and the assumed incidence of the other major osteoporotic fractures was determined from the Malmo ratios.

**Table 4:** The annual incidence (/100,000) of forearm and humeral fractures in women predicted from the epidemiology in Malmo (see methods) and that observed in the present study with 95% confidence intervals (CI)

Age (years)	Forearm			Humerus		
	Predicted	Observed	95% CI	Predicted	Observed	95% CI
Men						
50–59	298	229	162–315	113	109	64–172
60–69	286	206	126–318	103	154	85–255
70–79	85	78	16–226	128	52	6–187
80–89	79	165	20–598	100	0	0–305
Women						
50–59	516	**804**	**689–934**	148	164	114–228
60–69	497	**673**	**546–821**	188	**271**	**192–370**
70–79	640	743	556–971	332	294	182–450
80–89	507	748	457–1156	338	**599**	**342–972**

The observations in bold denote a significant difference between observed and predicted estimates

**Fig. 1:**
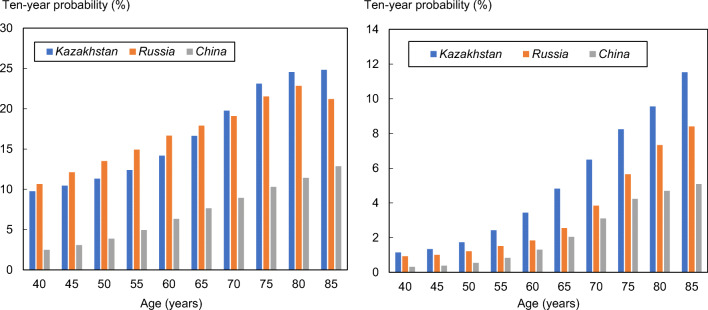
Ten-year probability of a major osteoporotic fracture (left hand panel) and hip fracture (right) in women with a prior fracture by age from Kazakhstan, Russia and China. Body mass index set to 25 kg/m^2^

The 10-year probability of major osteoporotic fracture and hip fracture in Kazakhstan and neighbouring countries is shown in Fig. 1 in women with a prior fracture by age. Ten-year probabilities were consistently higher than in the neighbouring country of China. In the case of Russia, 10-year probabilities of a major fracture were similar to those of Kazakhstan, but for hip fracture, the probabilities in Russia were substantially lower than those in Kazakhstan.

Lifetime probabilities for hip fracture are shown in Table 5. As it was the case for 10-year probabilities, lifetime probability of hip fracture was higher than that of Russians or Chinese but substantially lower than rates in Western Europe and North America.

**Table 5:** Life-time probability of hip fracture in the Kazakh population from the age of 50 years compared with selected countries

Country	Life-time risk at 50 years %	
	Women	Men
Sweden	25.6	11.0
Denmark	23.0	11.3
France	19.3	5.9
China (Hong Kong)	17.7	7.6
USA (Caucasian)	16.1	7.5
Turkey^a^	15.9	3.6
Canada	15.5	5.8
Greece	15.4	6.8
UK	14.4	5.0
Portugal	13.7	4.8
Finland	12.9	6.0
Kazakhstan^b^	12.6	6.0
Spain	12.6	4.2
Bulgaria	11.2	4.4
Hungary	10.8	4.2
Mexico^c^	10.6	5.0
Poland^d^	10.1	4.2
Moldova^e^	9.3	5.7
Russia^f^	7.7	3.8
Serbia	7.6	3.7
Romania^g^	7.1	3.8
China	5.9	3.3
Ukraine^h^	5.6	2.9

^abcdefgh^From [20] except where indicated; Tuzun et al. 2011 [21]; This study; Clark et al., 2005 [22]; Czerwinski et al., 2009 [23]; Zakroyeva et al. 2019 [12]; Lesnyak et al. 2012 [9]; Grigorie et al. 2013 [24]; Povoroznyuk et al. 2017 [25]

## Discussion

This study documented the incidence of hip, distal forearm and proximal humeral fractures in a region of Kazakhstan. As expected, hip fractures were more frequent in women than in men (female/male ratio = 1.6). In both sexes, the incidence increased with age. It is of interest that for individuals younger than 70 years, the hip fracture rate among men was slightly higher than in women. Thereafter, incidence was higher in women. Similar results have been reported in several studies [24, 26–28] including other countries of the EVA project, namely Armenia [10], Belarus [11], Moldova [12] and Russia [9]. Assuming that the regional incidence was similar to the national incidence, Kazakhstan belongs to the moderate-risk countries for hip fracture for men and women [29].

The number of hip fractures nationwide was estimated at 11,690 in 2015.

Demographic projections indicate that the annual number of hip fractures will increase by 140% to 28,048 in 2050. These estimates are relatively robust in that all individuals who will be aged 60 years, or more in 2050 are currently adults. However, these estimates may be conservative since they assume that the age- and sex-specific risk of hip fracture remains unchanged over this period. If the age- and sex-specific incidence of hip fracture increases, as has been registered in several countries [30], then the number of fractures may be more than doubled. Such projections are important for healthcare planning.

The access to all medical records in this study, including those from primary care, permitted the identification patients with hip fracture who were not admitted to hospital. The reason for this strategy was the observation that many patients in Eastern Europe are not hospitalized because facilities for surgical management are limited so that hospital admission is not feasible. In Belarus, for example, 29% cases of hip fracture did not come to hospital attention [11]. High rates of non-admittance have been reported in Armenia (44%) [10], Pervouralsk in Russia (27%) [9], Georgia (75%) and Kyrgyzstan (50%) [31]. The present study indicated that 25% of hip fracture cases were not admitted to hospital, and 43% of hip fracture cases were either untreated or managed conservatively. The treatment gap arises for many reasons including a lack of emergency orthopaedic surgeons. These findings are important for healthcare planning; they also emphasize the importance of exploring care pathways in the design of epidemiological studies.

A minority of countries that have a FRAX model also have robust information on the risk of other major osteoporotic fractures. In the absence of such information, FRAX models are based on the assumption that the age- and sex-specific pattern of these fractures is similar to that observed in Malmo [16]. This assumption has been shown to be safe in studies reported from Canada [18], Iceland [17], the USA [32], the UK [33], Australia [34] and Moldova [12], despite the differences in incidence between these countries [29]. This commonality of pattern is supported by register studies, which indicate that in those regions where hip fracture rates are high, so too is the risk of forearm fracture and spine fractures (requiring hospital admission) [35–37].

The acquisition of data on the incidence of forearm and humerus fractures as well as for hip fracture permitted the adequacy of this assumption to be tested in the present study, at least for forearm and humeral fractures. Our findings suggest that the incidence of forearm and humerus fractures can be reasonably predicted from the incidence of hip fracture in men. In women, however, the observed fracture rates exceeded those predicted from the Malmo ratios, in some cases significantly so. This disparity may arise because humeral and distal forearm fractures are relatively more common than hip fractures in women from Kazakhstan than in other counties. Unexpectedly, high rates of forearm and humeral fractures have been reported in Russia [9] and Hungary [27]. An alternative explanation is that not all cases of hip fracture were identified, particularly in women. The present study could not address the alternatives.

The incidence of hip fracture was used to create a FRAX tool to compute the 10-year probabilities of hip and major osteoporotic fracture in Kazakhstan. Ten-year probabilities were consistently higher than in the neighbouring country of China but for major osteoporotic fractures similar to that reported for Russia.

The widespread availability of FRAX has resulted in its adoption in many practice guidelines worldwide [7]. The fracture probability equivalent to a woman with a prior fracture has been used as an intervention threshold in more than 30 countries. If the same threshold were applied to Kazakhstan, then intervention would be recommended with a probability of a major fracture that varied between 9.7 and 25% depending on age (see Fig. 1). The impact of such thresholds or alternative thresholds will require further study.

There are a number of additional limitations to this study. With regard to fracture incidence, we examined only about 1% of the Kazakh population from a single centre. Therefore, the extrapolation of this regional estimate to the entire country is an assumption that we were unable to test. In addition to large variations in fracture rates around the world, fracture rates may vary within countries. In addition to ethnic-specific differences [38], up to two-fold differences in hip fracture incidence have been reported using common methodology with the higher rates in urban communities including Croatia [39], Switzerland [40], Norway [41], Argentina [42] and Turkey [43]. No distinction was made in the level of trauma. However, the division between high and low trauma is problematic in that osteoporotic patients fracture more commonly than non-osteoporotic patients following high trauma [44, 45]. Additionally, BMD is similar in patients with hip fracture, irrespective of the level of trauma [46]. These data support the inclusion of high-trauma fractures in epidemiological assessment.

As noted above, it is possible that not all hip fractures were captured, an effect that would give rise to a systematic underestimate of fracture probabilities for both hip fracture and major osteoporotic fracture. It is relevant, however, that accuracy errors have little impact on the rank order with which the FRAX tool categorizes risk in a given population [10, 25, 47], but they do change the absolute number generated, and thus have implications where treatment guidelines are based on cost-effectiveness or the economic burden of disease.

In summary, a FRAX model has been created for the Republic of Kazakhstan that based on a regional population-based estimate of the incidence of hip fracture. The model should enhance accuracy of determining fracture probability among the Kazakh population and help to guide decisions about treatment.
